# Invasive plants and their root traits are linked to the homogenization of soil microbial communities across the United States

**DOI:** 10.1073/pnas.2418632121

**Published:** 2024-10-24

**Authors:** Gabriela C. Nunez-Mir, Matthew A. McCary

**Affiliations:** ^a^Department of Biological Sciences, University of Illinois, Chicago, IL 60607; ^b^Department of Bioscience, Rice University, Houston, TX 77005

**Keywords:** functional traits, invasion, biotic homogenization, soil microbes, macroscale

## Abstract

Although the impacts of invasive plants on soil ecosystems are widespread, the role and impacts of invader root traits in structuring microbial communities remain poorly understood. Here, we present a macroecological study investigating how plant invaders and their root traits affect soil microbial communities, spanning data from 377 unique plots across the United States sampled multiple times, totaling 632 sampling events and 94 invasive plant species. We found that native and invasive plants harbor different root traits on average, with invasive plants possessing higher specific root lengths and native plants having higher root tissue density. We also show that soil microbial communities experiencing heavy plant invasions were more similar to each other in composition across ecosystem types and geographical regions than plots with higher proportions of native plants, which displayed highly variable microbial communities across the continent. Root traits of invasive plants in highly invaded plots explained two times more variation in microbial composition than native plants. This work represents an important step toward understanding macroscale and cross-scale patterns of the relationship between plant invasions, root traits, and soil microbial composition. Our findings provide insights into how invasive plants may impact ecosystem functioning at the macroscale via their homogenizing influence on microbial communities.

## Significance

Invasive plants can have strong, cascading effects on recipient ecosystems. Using a functional trait framework has been useful in understanding the mechanisms underlying these invasion impacts. However, belowground traits of invasive plants have been largely overlooked in the study of invasive impacts in favor of more easy-to-measure aboveground plant traits. Our macroecological study of belowground traits finds that not only are the roots of invasive plant species different from those of native plants, but that invasive plant roots alter the composition of soil microbial communities, explaining twice as much variation in microbial composition than native plants. We also find that invasive plant roots are associated with homogenous microbial communities across the United States, with important implications for ecosystem functioning.

The introduction of invasive plants into novel ecosystems, a phenomenon that has increased precipitously in recent decades (1), is one of the primary drivers of anthropogenic global change (2, 3). The impacts of invasive plants on recipient ecosystems can be wide-reaching, from eroding native biodiversity, altering ecosystem functioning and processes, to restructuring community composition (4–7). Thus, a major goal of ecological research is to characterize the impacts of invasive plants on native ecosystems and elucidate the mechanisms underlying their effects (8, 9).

A rich body of research has attempted to untangle the complex effects that invasive plants have on soil microbes and associated soil properties (10–13). Soil microbes (fungi, bacteria, archaea) are essential to maintaining terrestrial nutrient cycling (14) and serve as foundations of soil food webs (15, 16). Hence, resolving how plant invaders impact microbial communities is crucial for appreciating their full impact on native ecosystems. However, previous research suggests seemingly conflicting relationships between invasive plants and soil microbes (17–19), making it difficult to generalize invader impacts on recipient microbial communities. For example, a global meta-analysis exploring the effects of invasive plants indicated that invaded soils displayed enhanced microbial biomass when compared to uninvaded soils (7). In contrast, recent studies suggest that invasive plants can strongly reduce microbial biomass (20, 21), shift microbial composition (e.g., beta diversity) (22), have no effects (23), or that invasive plant effects are species-specific (19, 24). Inconsistent patterns in microbial responses to plant invasion may result from variability in the root traits displayed by different invasive plants and their unique interactions with the recipient ecosystem (6, 19, 25). In recent years, there has been an emphasis on adopting a functional trait framework to investigate the drivers and impacts of biological invasions, particularly for invasive plants (26–28). However, much of what is known about functional traits in relation to plant invasions is limited to functional traits that are easily observed in the aboveground context, such as shoot biomass, leaf C:N ratios, leaf area, and shoot growth rate (28–30). While aboveground functional traits have been valuable for understanding the impacts of plant invaders on recipient ecosystems, the body of literature on invasive plants has overlooked the traits that occur belowground.

Plant root traits are likely critical for determining the impacts of invasive plants on soil microbes, given that many invaders are successful partly due to their ability to rapidly reproduce or spread via root clones, utilize a variety of root exudates to defend against antagonists, and/or recruit microbial symbionts (7, 13, 25). These root traits likely elicit strong effects on soil microbial communities. For example, the widespread North American plant invader, garlic mustard (*Alliaria petiolata*), possesses a functional trait (i.e., allelopathic glucosinates) that suppresses the growth, diversity, and abundance of mycorrhizal fungi (31, 32)—an advantage linked to garlic mustard’s invasion success (33, 34). Similarly, autumn olive (*Elaeagnus umbellata*), a nitrogen-fixing shrub, can significantly alter the composition of bacterial and archaeal communities in the surrounding rhizosphere of native plants (35). Despite the well-known effects of these functional root traits on soil microbes (i.e., allelopathy or nitrogen-fixation), other root traits (e.g., root density, mycorrhizal colonization, root C:N ratio, etc.) might also affect the soil environment if these traits are distinct from those of native plants. However, almost nothing is known about the roles of these invasive root traits in modulating soil microbial communities. Because root traits like root C:N ratio, depth, and density could potentially shift the composition of microbial communities to favor certain taxonomic groups (e.g., by increasing bacterial dominance), decrease microbial diversity (i.e., decrease alpha diversity), or homogenize microbial communities (i.e., decrease beta diversity), it is crucial to understand how plant invaders’ root traits affect microbial communities across regions and ecosystems.

Here, we examine the underexplored relationship between root traits of invasive plants and the composition of soil microbial communities through a macroecology lens. Research on invasion phenomena, particularly research focused on predicting the effects of invasive species, can benefit greatly from a macroecological perspective, as this allows for the capture of widespread geographic patterns and the synthesis of contrasting patterns across scales, yielding insightful generalizations (36, 37). We conducted a macroscale analysis involving 693 native and 94 invasive plant species across 632 plot sampling events throughout the United States. The goal of this study was to investigate three questions: 1) Whether the roots of invasive and native plants are different? 2) How do the microbial communities of invaded and uninvaded plots differ? 3) Are root traits contributing to invasive impacts on microbial communities?

## Methods

### Study Area.

**Fig. 1.**
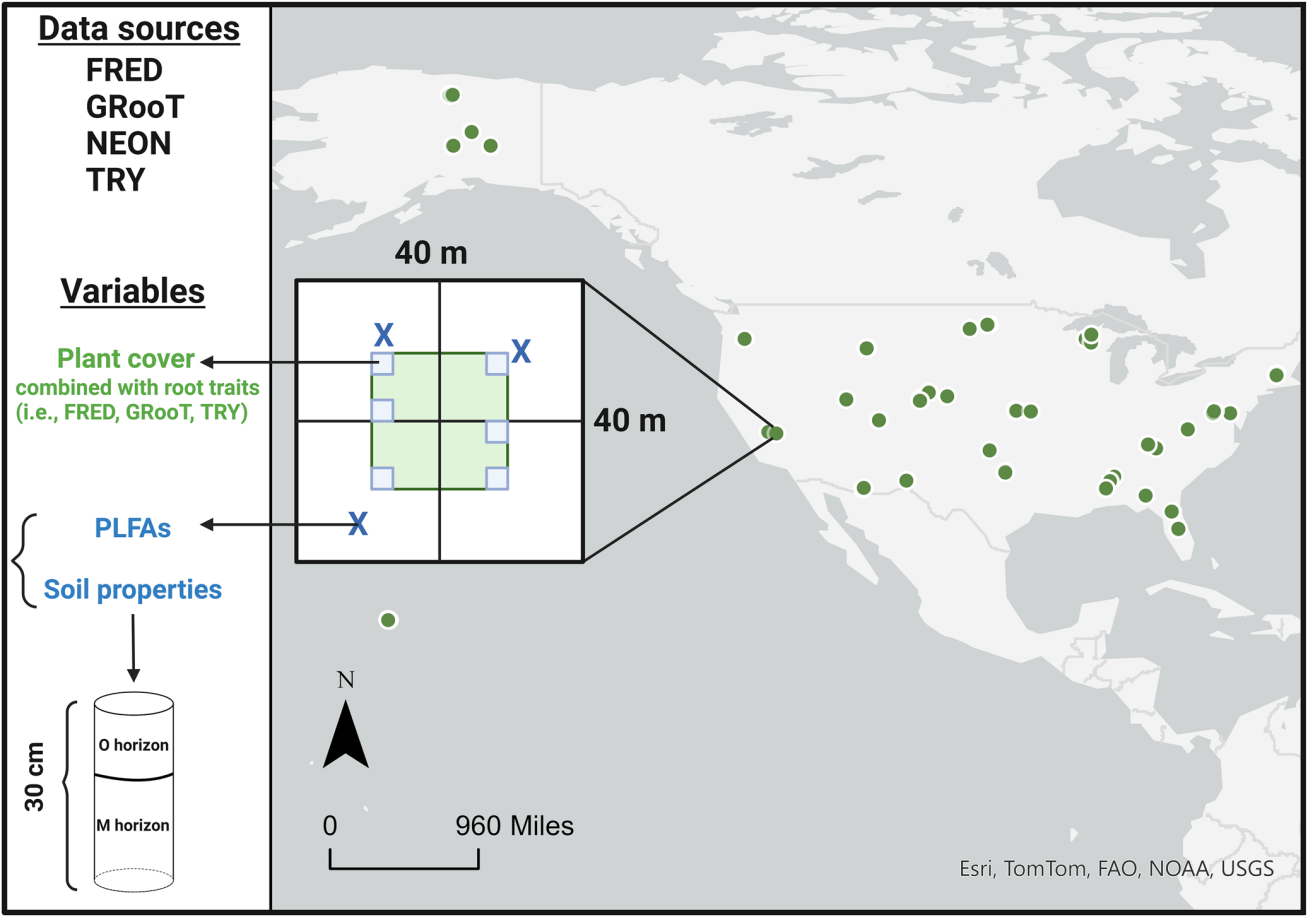
Schematic illustrating how the data were collected and organized across the United States, using data sources from NEON, FRED, TRY, and GRooT databases. The sampling design for the plants (small light blue boxes) and microbes and soil properties (dark blue “X” symbols) are provided; each plot (n = 377 unique plots spanning 42 terrestrial NEON sites) was 40 × 40 m in size.

Our study was performed using data from the United States NSF’s National Ecological Observatory Network (NEON). NEON is a continental-scale network designed to collect long-term and open-access ecological data to understand how ecosystems are changing in the United States. Here, we identified 377 unique NEON plots within the United States in which both vegetation composition and soil microbial data were sampled in the same year. The plots (40 × 40 m) that met these criteria spanned 42 terrestrial NEON sites across all ecoclimatic domains (excluding Atlantic Neotropical in the southern tip of Florida and Puerto Rico) (Fig. 1). These plots included diverse habitat types, such as managed (e.g., agroecosystems) and natural ecosystems (e.g., forests and grasslands) (*SI Appendix*, Table S1). Some plots were sampled for both plants and soil microbes over multiple years, resulting in a dataset of 632 sampling events from 2017 to 2021.

### Abundance and Root Traits of Invasive Plants.

To identify invasive plant species present in each plot and their abundance, we used the NEON data product “Plant Presence and Percent Cover” (Product ID DP1.10058.001) (38). Plant species presence and percent cover were documented in six 1-m^2^ subplots within each NEON plot (40 × 40 m). We aggregated the subplot data to obtain a species list for each plot, including the percent cover of each species within the plot. We identified 2,062 plant species across the 632 sampling events (1,865 native, 187 invasive, and 10 noninvasive/naturalized, as classified in the NEON database).

Root trait data for these species were obtained from the TRY Plant Trait Database (39), Fine-Root Ecology Database (FRED) (40), and Global Root Traits Database (GRooT) (41). We focused on nine root traits: 1) root diameter, 2) percent mycorrhizal colonization, 3) root C concentration, 4) root N concentration, 5) root C:N ratio, 6) specific root length, 7) root mass fraction, 8) rooting depth, and 9) root tissue density (*SI Appendix*, Table S2). These traits were selected for their ability to modulate soil microbiota through changes to the physical and chemical environment, as well as their data availability.

Despite considerable data collection efforts from the databases mentioned above, root trait data for plants are still relatively incomplete. Therefore, we proceeded with a data imputation method to address missing values in our dataset. Previous studies indicate that imputation in studies using incomplete trait data often outperforms analyses where only a subset of the data is used (42–44). We employed the “Rphylopars” R package, a tool to impute missing trait data via the estimation of phylogenetic and trait covariance across species (45). Before performing imputations, we followed the data cleaning procedures and best practices detailed in Johnson et al. (42). Data availability varied by trait analyzed. The most data-rich trait in our dataset was specific root length, with primary data for 46% of the species in our analyses (n = 787). The most data-deficient was root C concentration, representing 20% of primary data for the species in our analyses. We removed species for which we lacked data for the entire genus and species for which we only had data for 2 traits or fewer. We created a phylogenetic tree for all remaining species in our dataset using “U.PhyloMaker” R package (46). Our resulting trait dataset after imputation with Rphylopars contained complete data for 793 species (693 native, 94 invasive, and 6 noninvasive/naturalized). Given the small number of noninvasive/naturalized species, we excluded them from further analyses (*n =* 787). For each trait, we removed imputed values that fell outside the range of original values to maintain consistency between the imputed dataset and original data, thereby minimizing potential imputation errors that might bias our results.

### Abundance of Soil Microbial Communities and Soil Properties.

To derive the soil microbial composition of the identified NEON plots, we used the latest (2017 to 2021) phospholipid fatty acid analysis (PLFA) data from the “Soil Microbe Biomass” NEON data product (Product ID DP1.101041.001) (47). The NEON PLFA data product includes a full suite of microbial lipid biomarkers which can be used to identify microbial functional groups and quantify microbial composition (48) (*SI Appendix*, Table S3), including fungi, gram-negative bacteria, gram-positive bacteria, and Actinobacteria (48, 49). Although PLFA profiling can only provide abundance data for microbial functional groups, it provides information that compares favorably to next-generation DNA sequencing methods (50). For our study, we used 62 microbial lipid biomarkers to represent the soil microbial community (*SI Appendix*, Table S3).

To analyze PLFAs within each plot, three randomly selected locations were used to sample the top horizons of the soil (up to 30 cm; the O and M horizons of soil), which were then combined into a composite sample to represent the microbial community at the plot level. Most biotic activities (90%) occur in the O and M horizons of soil (51), so these soil depths are appropriate for capturing the composition of soil microbes despite each site having varying soil depths. A chloroform-methanol extraction method of lipid biomarkers was then performed and analyzed against a reference standard using Gas Chromatography (47). For additional details on NEON’s data collection and analysis, please refer to the publicly available protocols associated with each data product (47).

Soil properties are also linked to plant invasions and soil microbial composition (52–54). Thus, to evaluate the relevance of soil properties relating to levels of plant invasion, root traits, and soil microbial composition, we collected data on the physical soil properties for each plot. The data were obtained from the NEON data product “Soil Physical and Chemical Properties, periodic” (Product ID DP1.10086.001) (55). The product features soil physical and chemical properties that characterize the soils at each NEON plot; the data were collected between 2017 and 2021, depending on the plot. We obtained data on the horizons relevant to the rhizosphere (i.e., the O and M horizons). The following soil properties were extracted: soil moisture, pH, percent N, percent organic C, and C:N ratio. However, in our initial analyses, only soil moisture proved to be an important environmental factor explaining patterns of plant invasion or soil microbial composition, while the other variables were unimportant. Hence, we only report the results on soil moisture.

Although the soil profiles and depths differed across sites, the microbial and soil data were all collected from the soil organic layer (up to 30 cm; the O and M horizons), meaning our study measured how root traits correlated with the microbial community and soil properties within the top layer of the soil. Standardizing soil sampling was impractical due to the varying depths at each of the 377 unique plots across the United States that spanned grasslands, forests, and agroecosystems. However, this is a common issue with continental-scale data across different ecotypes (56–59); thus, we refer to our results as the influence of root traits on microbial communities in the top horizon of the soil.

## Statistical Analyses

### Q1. Are the Roots of Invasive and Native Plants Different?

Our first question focused on assessing whether the roots of invasive and native plants differ across the selected nine root traits. We performed a suite of phylogenetic linear regression models for each root trait with origin (native or invasive) as the predictor. We employed these linear regressions using the R package “phylolm” (Version 2.6.2) to account for the nonindependence of species due to phylogenetic relationships (60). Root trait values were transformed to avoid violations of normality prior to these analyses. To address estimation uncertainty from Rphylopars imputation, we repeated these analyses with the dataset prior to imputation (i.e., complete cases analysis) and with two separate datasets that contained the lower and upper bounds of the 95% CI for these estimations (i.e., lower and upper bound sensitivity analyses).

### Q2. Are the Microbial Communities of Invaded and Uninvaded Plots Different?

We addressed how strongly invasive plants impact microbial communities by comparing the microbial composition of plots with only native plants to plots containing varying levels of invasive plant cover. Since the plant and microbial compositions of these plots may change across years, we classified all 632 sampling events of the 377 unique plots into 3 invasion levels: “highly invaded,” if invasive species occupied ≥50% total plot cover (n = 44), “moderately invaded,” if invasive species occupied <50% total plot cover but were still present (n = 244), or “uninvaded,” if the plot was occupied exclusively by native species (*n =* 304). Sampling events in which a plot displayed a combination of native and naturalized species, but no invasive plants, were removed from our analyses, totaling 591 sampling events across 5 y. Prior to all site-level analyses, we verified that the proportion of plots in each habitat type were balanced across invasion levels to ensure our results would not be confounded by an uneven distribution of invasive plants across habitats (*SI Appendix*, Table S1).

We intended to employ two tests to determine how invasive plants affect soil microbial communities: a test of equal multivariate dispersion and a permutational ANOVA (PERMANOVA). The first test assessed whether the dispersion of microbial composition differed across invasion levels with the purpose of determining whether there is homogenization of microbial communities in invaded plots, as well as to complete an assumption check for the second analysis. This first test was performed using the *betadisper* function in the R package “vegan” (Version 2.5.7) to calculate the mean Bray–Curtis distance to the spatial median (a more conservative measure than the centroid) (61). We used the PLFA biomarkers as the multivariate proxy for soil microbial composition and plot invasion level as the categorical predictor. To address issues of pseudoreplication, we only performed these analyses with a subset of our data containing only the most recent sampling events for each plot (*n* = 361).

For the second analysis, we intended to perform a PERMANOVA between invasion levels to determine how invasive plants affect soil microbial communities. However, the assumption check performed with the first test failed (see *Results* section), indicating that any findings from a PERMANOVA would be unreliable. As a result, we did not proceed with the second analysis.

### Q3. Are Root Traits Contributing to Invasive Impacts On Microbial Communities?

For our last question, we wanted to evaluate the extent to which differences observed in the microbial composition of plots representing different invasion levels are influenced by the root traits of invasive plants (i.e., as opposed to leaf traits or other invasion mechanisms). We addressed this question through two approaches. The first approach utilized constrained ordinations to assess the contribution of different root traits to variation in microbial communities in plots with both invasive and native plants. The second approach involved a longitudinal analysis testing the effects of invasive root traits on microbial composition in plots that had been sampled for two consecutive years. In both approaches, we used an “invasive trait signal” metric for each of the nine root traits in our dataset instead of a simple community-weighted mean (i.e., the mean value for the species in a community weighted by the abundance of each species) for each trait. This metric was defined as the product of the community-weighted mean value for each trait in a given plot multiplied by the percent cover of invasive plants in the same plot. We opted for this approach to take into account the relative abundance of invasive plants in the plot compared to native plants. On average, we were able to collect data for all nine traits for 60% of the plant cover in each plot. To maximize statistical power in our dataset, we performed the analyses with all plots regardless of data coverage.

To execute the first approach, we used Redundancy Analyses (RDAs) and variation partitioning to determine which root traits from native and invasive plants explained the most variance in soil microbial composition. Because we were interested whether invasive plants were more influential in determining soil microbial composition compared to native plants, we selected plots that had both invasive and native plants (invasive and native plant cover had to be ≥20% of the total plant cover). Furthermore, to avoid issues of temporal pseudoreplication since some plots were sampled multiple times through the years, we opted to take an average of microbial composition and plant traits across years (*n* = 38 total plots). Microbial communities were Hellinger-transformed to minimize the effect of extreme values and double zeroes in the taxon-by-site matrix (62); we log-transformed all root trait variables to avoid violations of normality. We tested the root datasets for multicollinearity by calculating the variance inflation factor (VIF) for each variable; variables that displayed a VIF > 10 were removed (62). Next, we used variation partitioning to differentiate the relative effects of invasive and native plant root traits in structuring the soil microbial community. Variance partitioning uses partial RDAs to calculate the amount of variation in community structure explained uniquely by each explanatory matrix (i.e., either the invasive or native root dataset), as well as the shared variance explained by both explanatory matrices (63, 64). For this analysis, only pure fractions can be tested for statistical significance (i.e., a *P*-value can be calculated only for the complete native or invasive fractions).

Following the variation partitioning analysis, we performed separate RDAs for the invasive and native plants using a stepwise selection procedure to identify the root traits explaining a significant proportion of variation in microbial composition for each dataset; any root trait with a *P*-value smaller than 0.1 were retained in the model. For these RDAs, we selected native plots that had no levels of invasion and compared those to invaded plots (invasion cover ≥20% of total plant cover), enabling us to determine whether soil microbial communities associate with different root traits in invaded versus uninvaded plots. Because there were many more uninvaded plots (*n* = 328) than invaded plots (*n* = 44), we selected a random set of uninvaded plots (i.e., 44 plots) to mirror the sample size of the invaded plots. Furthermore, we also included important covariates in our RDAs—spatial structure (i.e., we built a distance-based Moran’s Eigenvector Map and included relevant eigenvectors into our model) and climate (i.e., mean annual temperature and precipitation)—to account for the influence of regional environmental heterogeneity on soil microbial composition throughout the United States. To visualize the relationship between invasive and native plants’ root traits and soil microbial composition, we present RDA ordination plots with the retained variables from the best models overlaid on top. All analyses were performed using the “vegan” package in R version 4.2.3.

For the longitudinal analysis in our second approach, we first identified plots that had been sampled in two consecutive years and for which we had been able to calculate community-weighted means for all root traits (*n* = 51). For each plot we obtained delta values for the PLFA biomarkers and invasive root trait signal metrics for the nine root traits. Because root traits can be strongly associated with each other, we examined the correlation matrix of all trait signals before analyzing their effects further (*SI Appendix*, Fig. S1). We chose a subset of trait signals that minimized collinearity while keeping the signals of traits that were found to differ between invasive and native species from Question 1. Our final set of trait signals included root C:N ratio, specific root length, rooting depth, and root tissue density. We then performed a PERMANOVA to test the effects of changes in these root trait signals, caused by temporal fluctuations in the presence and abundance of invasive plant species, on changes in microbial composition. To perform this analysis, we calculated a Euclidean distance matrix, instead of a Bray–Curtis distance matrix, since the multivariate response in this analysis was delta PLFA values. We then used the *adonis2* (PERMANOVA; 999 permutations) function in the R package “vegan.” We also added soil moisture to this PERMANOVA as a covariate. R-squared values produced by *adonis2* were corroborated with variance partitioning to better understand the variation explained by each trait after accounting for the effects of all other variables in the model.

## Results

### Q1. Invasive and Native Plants Differ in Specific Root Length and Root Tissue Density.

**Fig. 2.**
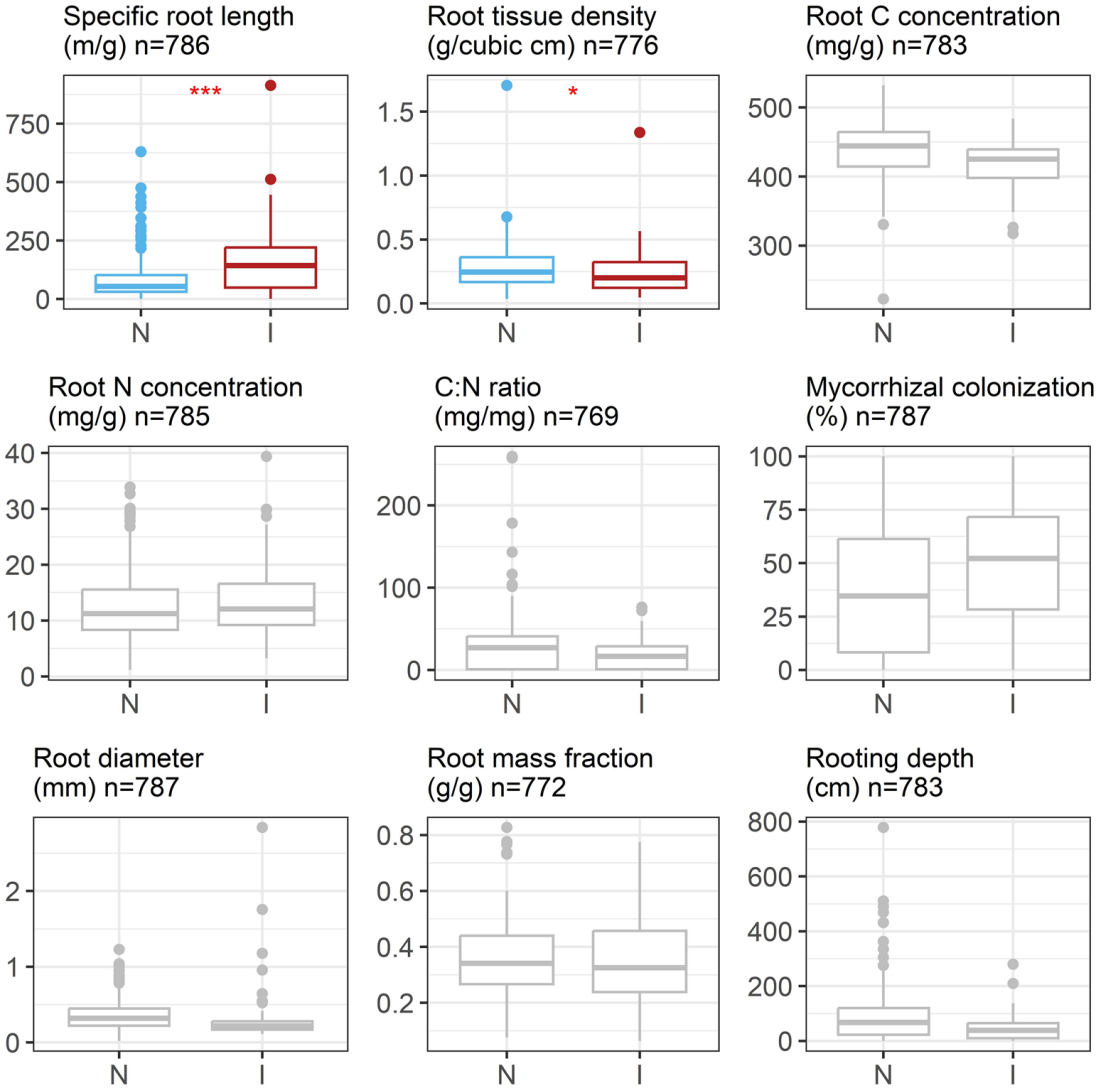
Boxplot comparison of nine root traits between native and invasive species using raw values for each species (i.e., prior to Box-Cox transformation). Boxplots illustrate root trait distribution with median values represented by the line inside the box, which spans the interquartile range (IQR), whiskers extending up to 1.5 times the IQR, and individual outliers displayed as dots. Red asterisks indicate statistically significant differences between native and invasive plant species according to our phylogenetic regression analyses (*** for *P* < 0.001; * for *P* = 0.029).

The results of our phylogenetic linear regressions using the imputed dataset showed that invasive and native plant species on average have significantly different specific root lengths and root tissue densities (Fig. 2). Invasive plants tended to have higher specific root length (β = 0.54, *P* < 0.001) and lower root tissue density (β = −0.13, *P* = 0.029).

Three additional analyses were performed to confirm the robustness of these results: one with the original traits dataset prior to imputation (i.e., the complete cases analysis), and two using datasets containing the lower and upper bounds of the 95% CI of imputed values (i.e., the lower and upper bound sensitivity analyses). Similar to the results obtained using the imputed dataset, specific root length was found to be strongly significant in the complete cases analysis (*SI Appendix*, Table S4) and the lower bound sensitivity analysis (*SI Appendix*, Tables S5 and S6) but not the upper bound sensitivity analysis. Root tissue density was found to be important in our complete cases analysis and the upper bound sensitivity analysis but not the lower bound sensitivity analysis. In general, the results of the complete case analysis were the same as the results presented here, while the sensitivity analysis found other traits to be different between invasive and noninvasive plants in addition to specific root length and root tissue density.

### Q2. The Microbial Communities of Invaded Plots Are More Homogenous than Those in Uninvaded Plots.

**Fig. 3.**
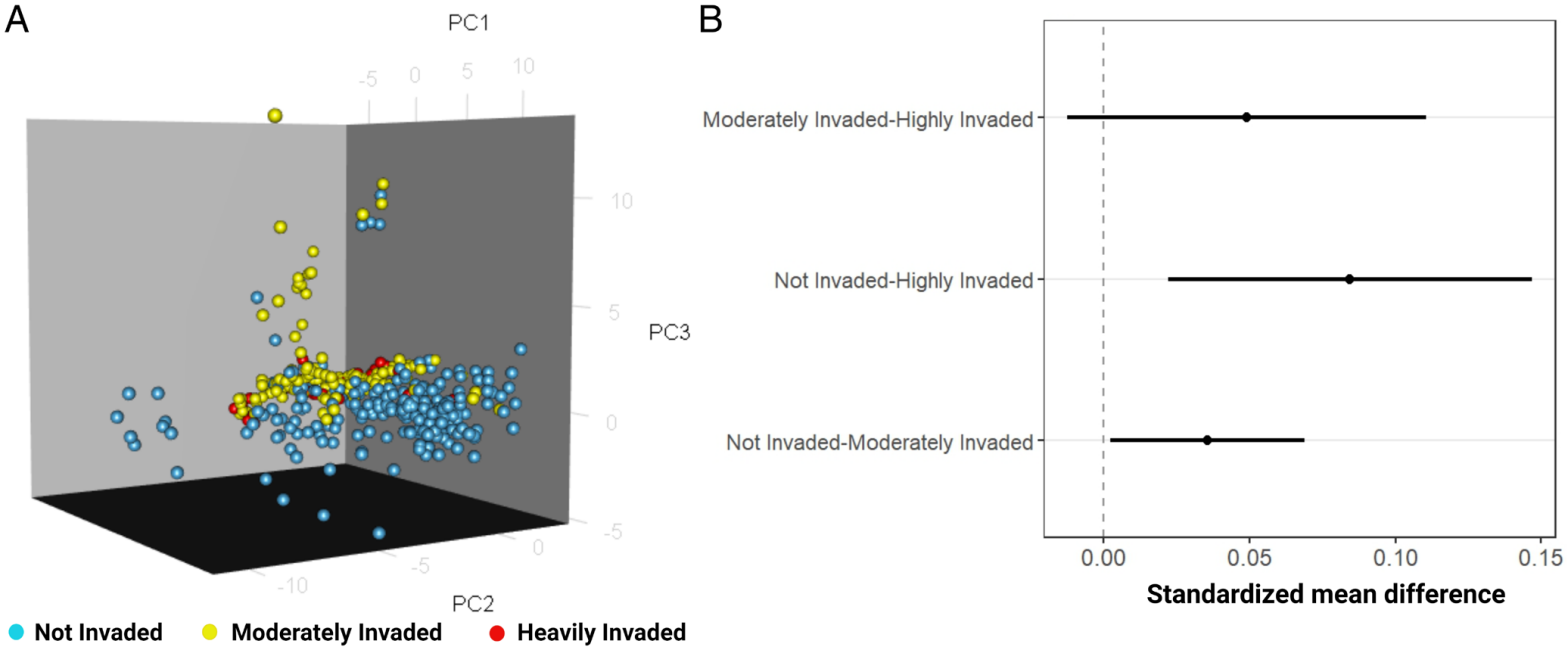
(*A*) Ordination showing how different levels of plant invasion affect the composition of soil microbial communities. (*B*) Tukey multiple comparisons of plot means with different invasion levels; error bars represent the 95% family-wise CI using all sampling events for each plot (*n* = 591). Highly invaded and moderately invaded plots were found to have significantly smaller dispersions than plots that were not invaded (Adj. *P* < 0.05).

Our test of multivariate dispersions showed that invaded plots, both highly and moderately invaded, had statistically significantly shorter Bray–Curtis distances to the spatial median (0.17 and 0.22, respectively) than uninvaded plots (0.26) (*F*_2, 359_ = 6.44, *P* < 0.002, Fig. 3*A*), indicating that invaded plots had more homogenous microbial communities than native plots. A Tukey HSD test demonstrated that invaded plots, both highly and moderately invaded, were significantly more different in dispersion level than the uninvaded plots (Fig. 3*B* and *SI Appendix*, Table S7).

### Q3. Invasive Root Traits Contribute to Observed Differences in Microbial Composition.

**Fig. 4.**
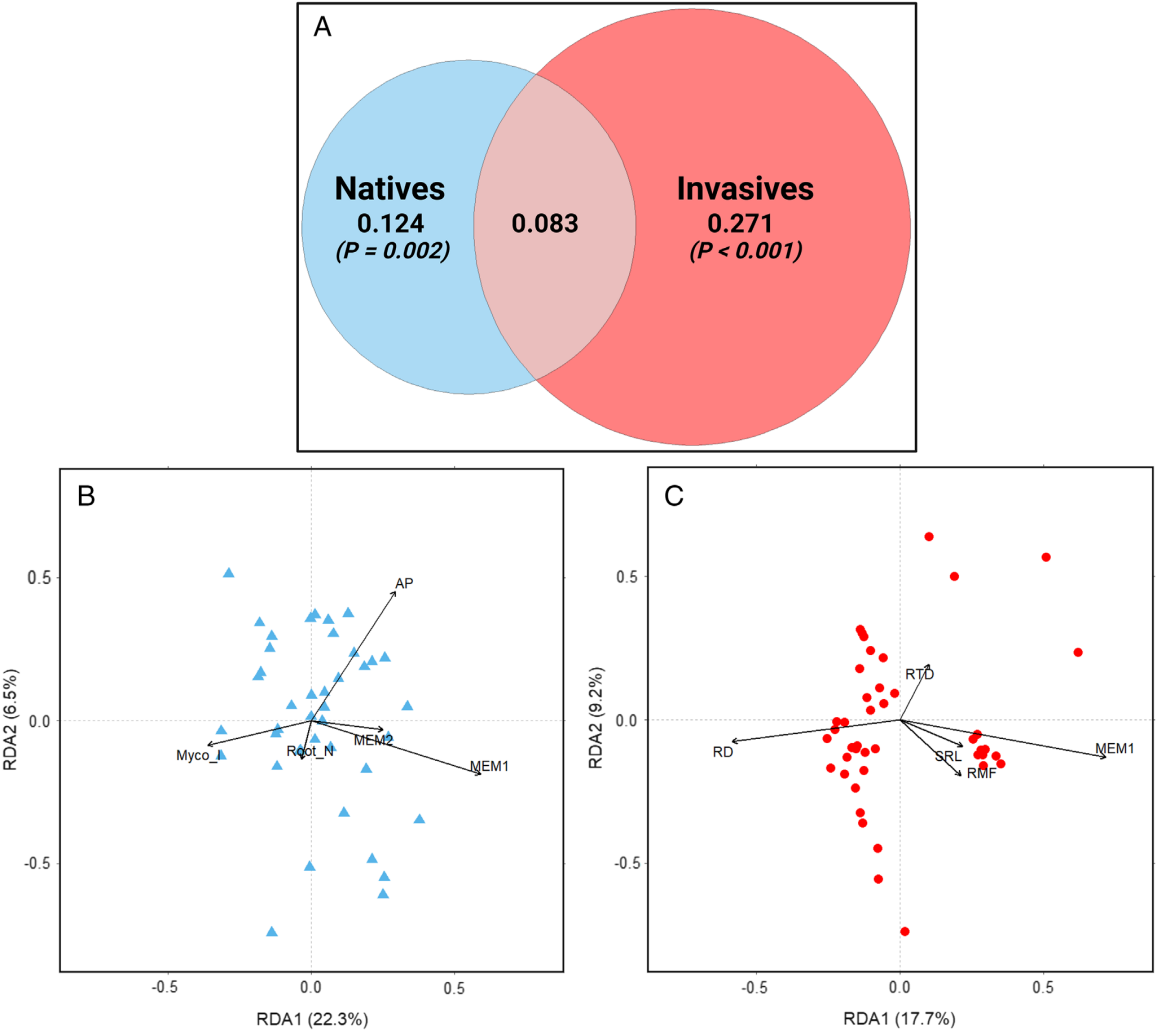
(*A*) Venn diagram illustrating the results of variation partitioning for variation explained by native and invasive plants in the same plots. Invasive plants and their associated root traits explained more than double the variation of microbial composition than native plants. RDAs with accompanying ordinations to show which root traits of (*B*) native plants and (*C*) invasive plants, respectively, explained the most variation in microbial composition and in what direction. Vector overlays depict the relationship of the root traits and the ordination axes, with the length and direction of each vector indicating the strength of relationship (Pearson’s correlation). AP = Annual Precipitation; MEM1 = Moran Eigenvector Map 1; MEM2 = Moran Eigenvector Map 2; Myco_I = mycorrhizal infection rate; SRL = specific root length; RD = rooting depth; RMF = root mass fraction; RTD = root tissue density; Root_N = root nitrogen content.

We found that the root traits of invasive plants explained twice as much variation in soil microbial composition compared to native plants, with the two trait datasets (i.e., invasive and native plants) combined explaining 48% of microbial composition (Fig. 4*A*). When examining which root traits were more influential for native plants (Fig. 4*B*) versus invasive plants (Fig. 4*C*) (i.e., separate RDAs), we found that invasive root traits of root mass fraction, specific root length, root tissue density, and rooting depth, as well as one of the spatial structure covariates (MEM1), were highly correlated with microbial composition (Table 1 and Fig. 4*C*); each of these traits was significantly associated with microbial composition. In contrast, when considering native plants, we found two root traits that were important: root N concentration and percent mycorrhizal infection (Fig. 4*B*). Three covariates were also found to be important for native plots: two spatial structure covariates (MEM1 and MEM2) and mean annual precipitation (AP). The invasive plant root data explained slightly more variation than the native plant data overall, with invasive and native plants explaining 27% and 25% of microbial composition, respectively.

**Table 1.** Root traits that potentially influence soil microbial composition

Variables	F	*P*-value
Native plants		
Root traits		
C:N ratio		
Mycorrhizal infection	3.62	0.015
Root C concentration		
Root diameter		
Root mass fraction		
Root N concentration	2.94	0.025
Root tissue density		
Rooting depth		
Specific root length		
Environmental variables		
AP	4.65	0.005
MAT		
MEM1	3.76	0.005
MEM2	2.37	0.035
Invasive plants		
Root traits		
C:N ratio		
Mycorrhizal infection		
Root C concentration		
Root diameter		
Root mass fraction	2.80	0.035
Root N concentration		
Root tissue density	3.57	0.005
Rooting depth	4.09	0.035
Specific root length	3.18	0.005
Environmental variables		
AP		
MAT		
MEM1	2.49	0.050
MEM2		

Open spaces indicate that the variable was not retained in the final model (*P* ≥ 0.10). The final model explained ~25% and 27% (adjusted R^2^) of microbial composition for native and invasive plants, respectively. AP = Annual Precipitation; MAT = Mean Annual Temperature; MEM = Moran Eigenvector Map.

In our longitudinal approach, our PERMANOVA found that changes in the invasive root signals of C:N ratio and specific root length in plots in consecutive years are significant predictors of changes in microbial composition (Table 2). C:N ratio explained the highest amount of variation among all explanatory variables in the model, accounting for just over 20% of the variation in community composition (*P* = 0.014), when taking into account the effects of all other variables. Specific root length had a weaker effect on microbial composition, explaining 6% of the variation (*P* = 0.045). Soil moisture, a covariate in the model, was also found to be statistically significant, explaining almost 16% of the variation in the data (*P* = 0.021).

**Table 2.** Results of a permutational ANOVA (PERMANOVA) testing the effects of delta values of root traits and soil moisture (as a covariate) on soil microbial composition (*n* = 51) and conditional adjusted R^2^ derived from variance partitioning analysis

Root trait	R^2^	F	*P-*value	Adj. R^2^
C:N ratio	0.195	15.14	0.012	0.204
Soil moisture	0.159	12.36	0.021	0.158
Specific root length	0.067	5.19	0.039	0.061
Root tissue density	0.006	0.49	0.370	−0.0076
Rooting depth	0.003	0.27	0.670	−0.01

## Discussion

Examining the ecosystem impacts of invasive plants and the mechanisms that generate them using a trait-based approach has provided valuable insights while lending a framework upon which generalizations and predictions could be formed (26, 65). However, previous studies have focused mostly on aboveground traits of invasive plants, leaving a substantial knowledge gap on how invasive plant root traits differ from native plants and how their traits affect soil microbial communities. With the first dataset of its kind, we performed a macroecological assessment of the impacts of invasive plants and their roots on soil microbial communities across the United States. Our study revealed that invasive plants generally have higher specific root lengths and lower root tissue density compared to native plants. These root trait differences explain more variation in microbial composition in invaded habitats, with invaded plots displaying homogenized soil microbial communities across geographic regions and ecosystems.

On average, invasive plants possess roots with higher specific root length but lower root tissue density compared to native plants. We also found that C:N ratio explained the highest amount of microbial variation across consecutive years of sampling. These root traits of plant invaders are consistent with faster root growth (i.e., low C:N ratios) and foraging capability (i.e., higher specific root lengths) (66), which many invasive plants are known to exhibit (65, 67). The presence of these root traits can modify the soil in ways that affect the growth of soil microbes. For example, in a study of 13 species of trees in subtropical China, researchers found declining abundances of soil bacteria (gram-negative and gram-positive) and fungi underneath trees with longer versus shorter specific root lengths (68). Invasive plants with high foraging abilities and growth rates compete with soil microbes for limiting nutrients (e.g., N and P) (69), thereby shaping microbial communities through their effects on nutrient availability and soil organic matter. Several studies have documented fast N uptake by plant root can strongly inhibit the growth of microbes in the rhizosphere (70, 71), potentially altering soil microbial community structure (72).

We also found the soil microbial communities were significantly more homogenous in plots with heavy plant invasions, providing evidence of biotic homogenization. Biotic homogenization is the process by which the genetic, taxonomic, and functional similarities among regional biological communities increase over time due to species invasions and their ensuing impacts, such as native species extirpations and environmental disturbances (73, 74). Our study suggests that invasive plants alter soil communities in similar ways, producing homogenous microbial communities regardless of ecosystem types and ecoregions. It is also worth highlighting the difficulty of detecting homogenization with coarse functional categories of microbes resulting from the PLFA. The microbial functional categories used in this study are ubiquitous (unlike the thousands of microbial species associated with DNA sequencing data), so observing homogenization at this level indicates that invasive plants have strong impacts on soil microbial communities.

The role of invasive species in the homogenization of native plant communities has been documented to a considerable extent (75–79). However, biotic homogenization of soil communities stemming from plant invasions is relatively unexplored. A recent study provided some evidence of an invasive plant obscuring latitudinal diversity patterns in coastal nematodes in China, leading to homogenized soil communities across latitudes (80). Other studies have reported that communities of soil fungal pathogens are similarly affected by plant invasions (e.g., *Alternanthera philoxeroides*), showing increased taxonomic similarity of soil fungal pathogens at local and regional scales, including across latitudinal gradients (81, 82). Such homogenization of soil communities (e.g., fungal communities) is likely to facilitate further plant invasions (83), with implications for terrestrial ecosystem structure and functioning (84).

While our study provides insights into how invasive plants and their root traits impact soil microbial communities across the United States, it is important to acknowledge the limitations of our macroecological approach that constrain the interpretation of our results. First, in our effort to assemble the most comprehensive dataset of plant root traits, vegetation composition and cover, soil microbial composition, and soil properties, we had to compromise the resolution of some data due to limited macroscale availability. As a result, we relied on PLFAs to characterize the microbial communities in our plots and species-level means for plant root traits. This approach, while necessary, may lead to an underestimation of the microbial responses at finer taxonomic resolutions and intraspecific variation in plant root traits across different environmental conditions. Such limitations may reduce the precision of our findings, potentially affecting the generality of our conclusions.

Second, we leveraged phylogenetic imputation to address missing values—a pervasive issue in plant trait databases (85), especially for belowground traits, which are notoriously difficult to collect. While a growing body of evidence suggests that analyses using imputed trait data outperform those using only incomplete original data, even the most advanced imputation methods introduce statistical uncertainty (42–44). To mitigate this, we supplemented our comparison of native and invasive root traits with two additional analyses: a complete cases analysis using only the original, incomplete trait data and a sensitivity analysis using the upper and lower bounds of the imputation estimates. The complete case analysis reinforced our confidence that the imputed data accurately reflects trait values, particularly in the context of comparing native and invasive plant species. The sensitivity analyses further support our findings; however, the statistical significance of additional traits in the sensitivity analyses suggests there could be additional traits (i.e., root mass fraction, mycorrhizal colonization, and root diameter) that are different between native and invasive plants. Because the imputation estimates for these traits were not precise enough to confidently assess these differences, we did not emphasize them in this article.

Given these limitations, our conclusions should be considered as an initial attempt to understand the role of invasive plant roots in homogenizing soil microbial communities. This represents a critical step forward, but further research is required to substantiate and expand upon these initial findings. A comprehensive understanding will require complementing our macroecological findings with localized field and greenhouse experiments. Future research should focus on analyzing these patterns at finer resolutions across various environmental and geographical contexts, characterizing microbial diversity using high-resolution taxonomic information (e.g., through Next-Generation DNA Sequencing) and directly measuring root traits. Such work would bridge the gap between localized and macroscale patterns, offering mechanistic insights into the scaling of these processes.

## Conclusions

The effects of invasive plants and their root traits on soil microbial communities have important implications for the functioning and health of terrestrial ecosystems. Common characteristics of invasive plant roots, such as higher specific root length and lower root tissue density, could structure the soil in ways that inhibit microbial growth while restructuring the soil microbial community. Our study provides initial evidence that invasive plant species share common root trait characteristics, which in turn may contribute to widespread homogenizing effects on soil microbial communities. Such homogenized microbial communities could result in the loss of other important functions at large spatial scales, such as decreased resilience to disturbance and loss of microbial mutualists and beta diversity. Moving forward, to obtain a more comprehensive understanding of plant invader impacts, subsequent research should characterize these potential effects at finer resolutions and more thoroughly investigate the mechanisms underlying these impacts via experiments. The potential for these impacts to have a positive feedback on new invasions should be also evaluated in future research.

## Supplementary Material

Appendix 01 (PDF)

## Data Availability

Access to all data and accompanying scripts are available on Github (https://github.com/mmccar26/invasive_roots_and_soil_microbes) (86). Previously published data were used for this work (38–41, 47, 55).
