# Supplementary material for: Invasive plants and their root traits are linked to the homogenization of soil microbial communities across the United States
Source: Proc Natl Acad Sci U S A. 2024 Oct 24;121(44):e2418632121. doi: 10.1073/pnas.2418632121 (PMC11536171; doi:10.1073/pnas.2418632121)
Supplement: Supplementary file 1 — Appendix 01 (PDF) [file pnas.2418632121.sapp.pdf]

PNAS

Supporting Information for

Invasive plants and their root traits are linked to the homogenization of soil microbial communities across the United States

Gabriela C. Nunez-Mir and Matthew A. McCary

Gabriela C. Nunez-Mir
Email: gnm@uic.edu

Matthew A. McCary
Email: mm160@rice.edu

This PDF file includes:

Tables S1 to S7
Figures S1

Table S1. Number of plots that have been classified as highly invaded, moderately invaded and not invaded across the six habitat types in our dataset.

**Habitat type**	***Highly Invaded***	***Moderately Invaded***	***Not Invaded***
Cropland	3 (7%)	19 (8%)	
Deciduous Forest	13 (29%)	76 (31%)	83 (27%)
Evergreen Forest	6 (14%)	35 (14%)	132 (43%)
Herbaceous	16 (36%)	63 (26%)	35 (12%)
Shrubland	3 (7%)	37 (15%)	33 (11%)
Wetland	3 (7%)	14 (6%)	21 (7%)
Total	44 (100%)	244 (100%)	304 (100%)

Table S2. Metadata on root traits included in our statistical analyses.

Variable	Unit	Category	Description
Root mass fraction	g/g	Morphology	Ratio of root dry mass per total plant standing dry biomass
Root N concentration	mg/g	Chemistry	Mass of N per root dry mass
Root C concentration	mg/g	Chemistry	Mass of C per root dry mass
Root C:N ratio	mg/mg	Chemistry	Ratio of carbon to nitrogen in root by mass
Mycorrhizal colonization intensity	Sum percent	Microbial associations	Percentage length of roots colonized by fungi
Specific root length	m/g	Morphology	Length of a root per unit dry mass
Root diameter	mm	Morphology	Mean diameter of the root sample
Root tissue density	g/cm^3	Morphology	Dry mass of root per unit volume of fresh root
Rooting depth	cm	System and distribution	Average soil depth at which roots occur

Table S3. Phospholipid fatty acid analyses (PLFA) functional designation for soil microbial communities. The PLFAs are classified according to the lipid C chain length; the functional designations are broadly defined (e.g., fungi or bacteria).

Lipid signature	Full name	Abbreviation	Biomarker	Functional group	Mean	SEM
aC15To0Concentration	12-methyltetradecanoic acid methyl ester, a-c15:0	anteiso C15:0	Gram-positive bacteria	Bacteria	5.278	0.707
c10To0Concentration	decanoate methyl ester, c10:0	C10:0	Bacteria, general	Bacteria	0	0
c11To0Concentration	undecanoate methyl ester, c11:0	C11:0	Bacteria, general	Bacteria	0	0
c12To0Concentration	dodecanoic acid, or lauric acid, methyl ester, c12:0	C12:0	Bacteria, general	Bacteria	0.237	0.05
c13To0Concentration	tridecanoic acid methyl ester, c13:0	C13:0	Bacteria, general	Bacteria	0.054	0.024
c14To0Concentration	tetradecanoic acid, or myristic acid, methyl ester, c14:0	C14:0	Bacteria, general	Bacteria	1.014	0.117
c14To1Concentration	cis-9-tetradecenoic acid, or myristoleic acid, methyl ester, c14:1	C14:1w9	Gram-negative bacteria	Bacteria	0.08	0.03
c15To0Concentration	pentadecanoic acid methyl ester, c15:0	C15:0	Bacteria, general	Bacteria	0.652	0.078
c15To1Concentration	cis-10-pentadecenoic acid methyl ester, c15:1	C15:1	Gram-negative bacteria	Bacteria	0	0
c16To0Concentration	hexadecanoic acid, or palmitic acid, methyl ester, c16:0	C16:0	Bacteria, general	Bacteria	11.91	1.486
c16To1Cis11Concentration	methyl cis-11-hexadecenoate, 16:1 cis 11	C16:1w11c	Gram-negative bacteria	Bacteria	3.606	0.568
c16To1n7Concentration	NA	NA	NA	NA	1.191	0.233
c17To0AnteisoConcentration	methyl 14-methyl-pentadecanoate, 17:0 anteiso	anteiso C17:0	Gram-positive bacteria	Bacteria	1.548	0.177
c17To0Concentration	heptadecanoic acid methyl ester, c17:0	C17:0	Bacteria, general	Bacteria	0.612	0.079

c17To1Concentration	cis-10-Heptadecenoic acid methyl ester, c17:1	C17:1	Gram-negative bacteria	Bacteria	0.108	0.033
c17To1n7Concentration	NA	NA	NA	NA	0.895	0.108
c18To0Concentration	octadecanoic acid, or stearic acid, methyl ester, c18:0	C18:0	Bacteria, general	Bacteria	1.895	0.331
c18To1n11Concentration	vaccenic acid or e-octadec-11-enoic acid, c18:1n11	C18:1w11	Algae	Algae	11.491	1.901
c18To1n13Concentration	NA	c18:1w13	NA	NA	1.123	0.19
c18To3n3Concentration	cis,cis,cis-9,12,15-octadecatrienoic acid, or alpha-linolenic acid, methyl ester, c18:3n3	C18:3w3	Fungi, general	Fungi	0.363	0.037
c18To3n6Concentration	6,9,12-octadecatrienoic acid, or gamma-linolenic acid, methyl ester, c18:3n6	C18:3w6	Saprophytic fungi	Fungi	0.093	0.064
c19To0Concentration	nonadecanoic acid methyl ester, c19:0	C19:0	NA	NA	0	0
c19To1Cis10Concentration	cis-10-nonadecenoic acid, methyl ester, c19:0	C19:1c10	NA	NA	3.062	0.769
c20To0Concentration	arachidic acid methyl ester, c20:0	C20:0	Bacteria, general	Bacteria	0.52	0.044
c20To1Concentration	cis-11-eicosenoic acid methyl ester, c20:1	C20:1	NA	NA	0.349	0.064
c20To2Concentration	cis-11,14-eicosadienoic acid methyl ester, c20:2	C20:2	Eukaryotic	NA	0.247	0.054
c20To3n3Concentration	cis-11,14,17-eicosatrienoic acid methyl ester, c20:3n3	C20:3w3	NA	NA	0.06	0.013
c20To3n6Concentration	cis-8,11,14-eicosatrienoic acid, or dihomo-gamma-linoleic acid, methyl ester, c20:3n6	C20:3w6	Protozoa	Protozoa	0.204	0.041
c20To4n6Concentration	cis-5,8,11,14-eicosatetraenoic acid, or arachidonic acid, methyl ester, c20:4n6	C20:4w6	Protozoa	Protozoa	0.759	0.197
c20To5n3Concentration	cis-5,8,11,14,17-eicosapentaenoic acid, or eicosapentaenoic acid, methyl ester, c20:5n3	C20:5w3	Algae	Algae	0.344	0.087
c21To0Concentration	heneicosanoic acid methyl ester, c21:0	C21:1	NA	NA	0.031	0.021
c22To0Concentration	docosanoic acid methyl ester, c22:0	C22:0	NA	NA	0.583	0.083
c22To1n9Concentration	cis-13-docosenoic acid, or erucic acid, methyl ester, c22:1n9	C22:1w9	Gram-negative bacteria	Bacteria	0.043	0.036
c22To2Concentration	cis-13,16-docosadienoic acid methyl ester, c22:2	C22:2	NA	NA	0.007	0.003

c22To6CisConcentration	cis-4,7,10,13,16,19-docosahexaenoic acid, methyl ester	C22:6	NA	NA	0.029	0.017
c23To0Concentration	tricosanoic acid methyl ester, c23:0	C23:0	NA	NA	0.114	0.035
c24To0Concentration	tetracosanoic acid, or lignoceric acid, methyl ester, c24:0	C24:0	NA	NA	0.696	0.117
c24To1Concentration	cis-15-tetracosenoic acid, c24:1	C24:1	NA	NA	0.052	0.024
c8To0Concentration	octanoate methyl ester, c8:0	C8:0	Bacteria, general	Bacteria	0	0
cis16To1n9Concentration	methyl hexadecenoic acid, or palmitoleic acid, methyl ester, c16:1n9	C16:1w9c	Gram-negative bacteria	Bacteria	5.964	0.981
cis18To1n9Concentration	cis-9-octadecenoic acid, or oleic acid, methyl ester, cis18:1n9	C18:1w9c	Cyanobacteria	Bacteria	6.54	1.092
cis18To2n912Concentration	cis,cis-9,12-octadecadienoic acid, or linoleic acid, or 18To2-omega-6, methyl ester, cis18:2n9-12	C18:2w9 12	Saprophytic fungi	Fungi	2.429	0.355
cyclo17To0Concentration	the cyclopropyl C17To0 fatty acid methyl cis-9,10-methylenehexadecanoate, cyclo17:0	cy17:0	Gram-negative bacteria	Bacteria	2.578	0.322
cyclo19To0Concentration	the cyclopropyl C19To0 fatty acid methyl cis-9,10-methyleneoctadecanoate, cyclo19:0	cy19:0	Gram-negative bacteria	Bacteria	7.427	1.222
i14To0Concentration	tridecanoic acid, 12-methyl-, methyl ester, iso-14:0	iso 14:0	Gram-positive bacteria	Bacteria	0.77	0.141
i15To0Concentration	13-methyltetradecanoic acid methyl ester, i15:0	i15:0	Gram-positive bacteria	Bacteria	7.442	0.906
i16To0Concentration	methyl 14-methylpentadecanoate, i16:0	i16:0	Gram-positive bacteria	Bacteria	2.906	0.293
i17To0Concentration	methyl 15-methylhexadecanoate, i17:0	i17:0	Gram-positive bacteria	Bacteria	1.458	0.197
lipid10Methyl16To0Concentration	methyl 10-methyl-hexadecanoate, 10 Me-16:0	10Me16:0	Actinobacteria	Bacteria	6.418	0.985

lipid10Methyl17To0Concentration	heptadecanoic acid methyl ester, 10 Me-17:0	10Me17:0	Actinobacteria	Bacteria	1.921	0.52
lipid10Methyl17To1Concentration	lipid 10 Methyl 17To1 Concentration	10Me17:1	Actinobacteria	Bacteria	0.997	0.119
lipid10Methyl18To0Concentration	tuberculostearic acid methyl ester, 10 Me-18:0	10Me18:0	Actinobacteria	Bacteria	1.819	0.191
lipid10Methyl18To1Concentration	lipid 10 Methyl 18To1 Concentration	10Me18:1	Actinobacteria	Bacteria	0.809	0.15
lipid2OH10To0Concentration	methyl 2-hydroxydecanoate, 2OH10:0	2OH10:0	Gram-negative bacteria	Bacteria	0.006	0.004
lipid2OH12To0Concentration	methyl 2-hydroxydodecanoate, 2OH12:0	2OH12:0	Gram-negative bacteria	Bacteria	0.475	0.113
lipid2OH14To0Concentration	methyl 2-hydroxytetradecanoate, 2OH14:0	2OH14:0	Gram-negative bacteria	Bacteria	0.351	0.124
lipid2OH16To0Concentration	methyl 2-hydroxyhexadecanoate, 2OH16:0	2OH16:0	Gram-negative bacteria	Bacteria	0.009	0.003
lipid3OH12To0Concentration	methyl 3-hydroxydodecanoate, 3OH12:0	3OH12:0	Gram-negative bacteria	Bacteria	0	0
lipid3OH14To0Concentration	methyl 3-hydroxytetradecanoate, 3OH14:0	3OH14:0	Gram-negative bacteria	Bacteria	0.177	0.036
totalLipidConcentration	Total lipid concentration calculated as the sum of all measured individual lipid components	Total lipids	None	None	93.358	12.215
trans18To1n9Concentration	trans-9-octadecenoic acid, or elaidic acid, methyl ester, trans18:1n9	C18:1w9t	Cyanobacteria	Bacteria	2.128	0.317
trans18To2n912Concentration	trans-trans-9,12-octadecadienoic acid, or linoelaidic acid, methyl ester, trans18:2n9-12	18:2w9 12t	Saprophytic fungi	Fungi	0.111	0.026

Table S4. Results of phylogenetic linear regressions comparing root traits of natives and invasive plants with the original dataset prior to imputation (i.e., complete cases analysis).

**Root trait**	***n***	**Estimate**	**SE**	***p***	
Root mass fraction	222	-0.009	0.054	0.87	
Root N concentration	286	-0.31	0.33	0.35	
Root C concentration	162	-7.19	8.97	0.42	
Root C:N ratio	300	-0.94	0.89	0.29	
Mycorrhizal colonization	195	-1.67	9.55	0.86	
Specific root length	365	1.05	0.25	< 0.001	***
Root diameter	300	0.03	0.11	0.77	
Root tissue density	259	-0.37	0.13	0.006	**
Rooting depth	239	-0.81	1.27	0.52	

Table S5. Results of phylogenetic linear regressions comparing root traits of natives and invasive plants with the dataset containing the lower bound of the 95% confidence interval for imputation estimates (i.e., sensitivity analysis).

**Root trait**	***n***	**Estimate**	**SE**	***p***	
Root mass fraction	515	0.31	0.11	0.004	**
Root N concentration	603	0.20	0.15	0.18	
Root C concentration	710	11.18	7.52	0.14	
Root C:N ratio	450	-0.20	0.60	0.73	
Mycorrhizal colonization	787	0.26	0.10	0.008	**
Specific root length	720	1.00	0.23	< 0.001	***
Root diameter	717	0.20	0.08	0.02	*
Root tissue density	599	0.05	0.15	0.73	
Rooting depth	627	0.01	0.29	0.96	

Table S6. Results of phylogenetic linear regressions comparing root traits of natives and invasive plants with the dataset containing the upper bound of the 95% confidence interval for imputation estimates (i.e., sensitivity analysis).

**Root trait**	***n***	**Estimate**	**SE**	***p***	
Root mass fraction	341	-0.05	0.05	0.29	
Root N concentration	503	-0.74	0.44	0.10	
Root C concentration	426	-4092.9	3550.7	0.25	
Root C:N ratio	395	-0.28	0.90	0.75	
Mycorrhizal colonization	787	-0.15	0.06	0.01	*
Specific root length	649	0.48	0.37	0.20	
Root diameter	665	-0.23	0.10	0.02	*
Root tissue density	488	-0.44	0.14	0.002	**
Rooting depth	458	-0.86	0.80	0.28	

Table S7. Results of Tukey multiple comparisons of means of plots with different invasion levels including 95% family-wise confidence interval using only most recent sampling events for each plot (*n* = 361).

	**Difference**	**Lower 95%**	**Upper 95%**	**Adj. *p***	
Moderately invaded-Highly invaded	0.05	-0.01	0.11	0.15	
Not invaded-Highly invaded	0.08	0.02	0.15	0.004	**
Not invaded-Moderately invaded	0.04	0.002	0.07	0.033	*

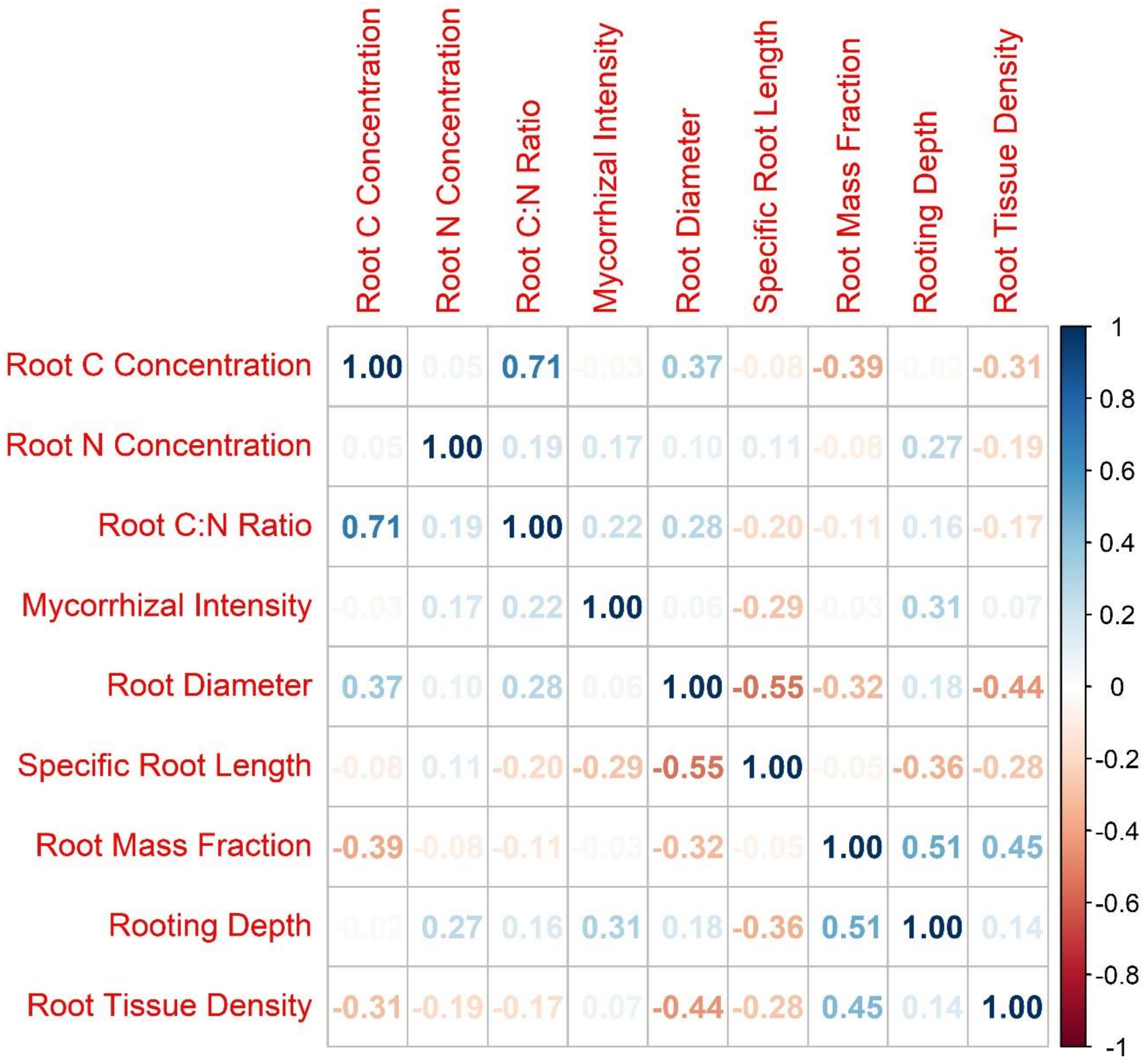

Figure S1. Correlation matrix of nine root traits. Values indicate Pearson's correlation coefficient for pairs of root traits using Box-Cox transformed trait values. Colors indicate the direction of the correlation.
